# Color Sensitivity of the Duration Aftereffect Depends on Sub- and Supra-second Durations

**DOI:** 10.3389/fpsyg.2022.858457

**Published:** 2022-03-22

**Authors:** Bingxin Lin, Youguo Chen, Li Pan, Gang Du, Xiting Huang

**Affiliations:** ^1^Faculty of Psychology, Southwest University, Chongqing, China; ^2^Center of Studies for Psychology and Social Development, Southwest University, Chongqing, China; ^3^Time Psychology Research Center, Southwest University, Chongqing, China

**Keywords:** duration aftereffect, color sensitivity, sub-second, supra-second, perception

## Abstract

The perception of duration becomes biased after repetitive duration adaptation; this is known as the duration aftereffect. The duration aftereffect exists in both the sub-second and supra-second ranges. However, it is unknown whether the properties and mechanisms of the adaptation aftereffect differ between sub-second and supra-second durations. In the present study, we addressed this question by investigating the color sensitivity of the duration aftereffect in the sub-second (Experiment 1) and supra-second (Experiment 2) ranges separately. We found that the duration aftereffect in the sub-second range could only partly transfer across different visual colors, whereas the duration aftereffect in the supra-second range could completely transfer across different visual colors. That is, the color-sensitivity of the duration aftereffect in the sub-second duration was stronger than that in the supra-second duration. These results imply that the mechanisms underlying the adaptation aftereffects of the sub-second and supra-second ranges are distinct.

## Introduction

The perception of time relates closely to many cognitive functions. For example, estimations of interval timing in the milliseconds-to-seconds range are important for motor control and language processing (Meck, 2005). However, the perception of duration is biased in some cases. Researchers have found that a duration aftereffect is induced by duration adaptation, similar to the tilt aftereffect and motion aftereffect (Heron et al., 2012; Li et al., 2015b). After repetitive exposure to a stimulus of relatively short duration, the subsequent stimulus is perceived to be of longer duration, and after repetitive exposure to a stimulus of relatively long duration, the subsequent stimulus is perceived to be of shorter duration.

There is an extensive body of research on the properties of the duration aftereffect. Findings have revealed that the duration aftereffect is sensory-specific (Heron et al., 2012), such as being contingent on auditory frequency but not on the visual orientation of a stimulus (Li et al., 2015b). Moreover, the region of spread of the duration aftereffect depends on the size of the adapting stimulus; the larger the adapting stimulus, the greater the spatial spread of the aftereffect (Fulcher et al., 2016). However, these studies examined duration in the sub-second range. Recent studies have found that duration aftereffects also exist in the supra-second range (Shima et al., 2016; Li et al., 2017b). Are the properties and mechanisms of the duration aftereffect in the supra-second range consistent with those found in the previous studies of the sub-second range? This is not yet known. Several studies have suggested differences in the mechanisms by which sub-second and supra-second durations are processed (Lewis and Miall, 2003a; Hayashi et al., 2014). Lewis and Miall (2003b) proposed the existence of automatic and cognitively controlled timing systems by analyzing previous neuroimaging studies of sub- and supra-second durations. The perception of a duration of less than 1 s is automatic, without being subject to attentional modulation. The “automatic” timing system is closely linked to the motor and premotor circuits. However, the perception of durations greater than 1 s requires attention and memory; thus, this represents a “cognitively controlled” timing system that draws heavily upon the prefrontal and parietal cortices. The features and mechanisms of the duration aftereffect in the sub-second range may not apply to the supra-second range, given the differences between the systems that process sub-second and supra-second stimuli.

Studies have found that visual areas are organized into two functionally specialized processing pathways: the dorsal and ventral streams (Ungerleider, 1995; Kastner and Ungerleider, 2000). The dorsal stream is crucial for spatial vision, which is necessary to appreciate the spatial relations among objects and guide movements toward objects in space. The ventral stream is important for object vision, which is responsible for identifying stimulus attributes such as shape, color, and texture. More importantly, Battellil et al. (2008) proposed both “where” (dorsal stream) and “what” (ventral stream) pathways, which play critical roles in perceiving the timing of visual events. However, most previous studies of the factors that influence the visual duration aftereffect focused on the dorsal stream such as by considering position (Li et al., 2015a; Maarseveen et al., 2017), size (Fulcher et al., 2016), and orientation (Li et al., 2015b). Few studies focused on the ventral stream. Therefore, we investigated the sensitivity of the duration aftereffect to color from the “ventral stream” perspective.

An unsolved problem is whether the neural underpinnings of duration adaptation in the sub-second and supra-second ranges are the same. Fortunately, investigation of the color sensitivity of the duration aftereffect, namely its specificity or invariance across different colors, may reveal the neural mechanisms underlying duration adaptation. This is because the primary visual cortex contains large populations of color-selective neurons (Engel and Furmanski, 2001; Nishida et al., 2003; Nieman et al., 2005; Parkes et al., 2009). The most color-selective neurons may relay color signals in V1, as opposed to later visual areas (Wachtler et al., 2003; Engel, 2005). Therefore, if the duration aftereffect exhibits strong color-sensitivity, this could be explained as the sub-second duration adaptation involving primarily the early visual cortex; conversely, weak color-sensitivity would suggest greater involvement of later visual cortical regions. Due to differences in processing mechanisms between the sub-second and supra-second durations, we expected the sub-second duration aftereffect to exhibit relatively strong color-sensitivity and the supra-second duration aftereffect to exhibit relatively weak color sensitivity. In the present study, we designed two experiments to investigate whether the sub-second/supra-second duration aftereffect could be transferred across different visual colors to verify whether the duration aftereffects in the sub-second and supra-second ranges have distinct mechanisms. In the first experiment, the subjects adapted to a visual stimulus (white disk) with a given duration (sub-second), which was presented in the center of the screen. The subjects were then tested with disks of random colors (white or red) at the same position. The goal of Experiment 1 was to evaluate whether the sub-second duration aftereffect could transfer across different visual colors. The design of the second experiment was similar to that of the first experiment, except that the durations of adapting stimuli and test stimuli were in the supra-second range. The goal of Experiment 2 was to evaluate whether the supra-second duration aftereffect could be transferred across different visual colors. This study examined whether stimulus duration can affect the color sensitivity of the duration aftereffect to explore whether the mechanism underlying the duration aftereffect is different between the sub-second (Experiment 1) and supra-second (Experiment 2) durations.

## Experiment 1

The aim of Experiment 1 was to investigate the color sensitivity of the duration aftereffect in the sub-second range.

### Materials and Methods

#### Participants

Twenty participants (eight men, mean age: 21.85 ± 1.81 years) were unaware of the experimental purpose. All participants provided written informed consent, which was approved by the Local Ethics Committee of the Southwest University of China and was conducted in accordance with the Declaration of Helsinki. All participants were right-handed and had normal or corrected-to-normal vision.

#### Stimuli and Apparatus

The visual stimulus was a white or red disk (0.5°), presented at the center of a CRT monitor (100 Hz refresh rate, 1,024 × 768 pixels), with a gray background (12.8 cd/m^2^). The participants were seated at a viewing distance of approximately 70 cm. E-Prime 2.0 software (Psychology Software Tools, Inc., Pittsburgh, PA) was used to control the presentation and the stimuli’s timing, and to record the data.

#### Procedure

**Figure 1:**
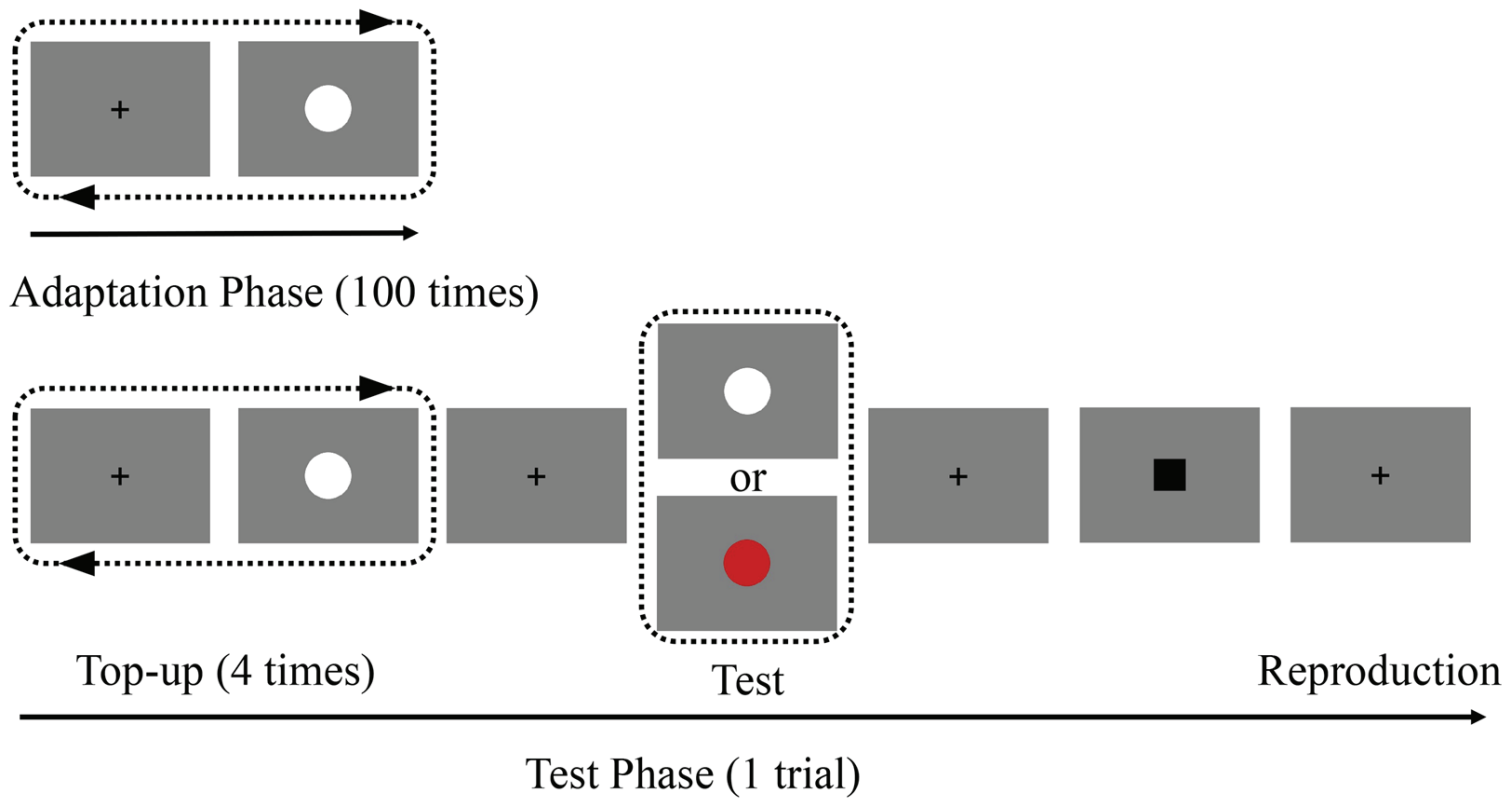
Schematic representation of the experiment. In the adaptation phase, participants viewed 100 repetitions of the adaptation stimulus (white disk; 200 or 800 ms) in the center of the screen. In the subsequent test phase, participants were asked to press the “ENTER” key to reproduce the duration of the test stimulus when the black square appeared. The colors of the test stimuli were presented randomly.

Experiment 1 consisted of four blocks, each of which contained an adaptation phase and a test phase (Figure 1). In the adaptation phase, participants adapted to a white disk, which was serially presented 100 times. Within each block, the duration of the adaptation stimuli was fixed at either 200 or 800 ms. The inter-stimulus interval was randomly jittered between 500 and 1,000 ms, while the test phase followed a short pause. During the test phase, each trial started with four top-up adapting stimuli (white disk; the same duration as the adaptation phase) followed by a test stimulus (white or red disk). There was a 2,000-ms pause between the final top-up adaptor and the test stimulus. That is, the colors of the adapting stimuli and test stimuli were either the same (white) or different (red). To reduce the noise associated with the responses, a pause was randomly jittered between 500 and 1,000 ms after the test stimuli. A black square (0.40 × 0.40°) was then displayed to remind the participants to prepare for the reproduction of the duration. Once the black square appeared, participants were required to press the “ENTER” key, which was held as accurately as possible for the duration of the test stimulus. During each block, the durations of the adapting and top-up stimuli were identical and fixed. However, the test stimulus durations varied randomly according to the following distribution: approximately 80% lasted 500 ms, approximately 10% lasted 300 ms, and the remaining 10% lasted 700 ms. Trials that lasted 300 ms and 700 ms were adopted to avoid the participants from duplicating the duration through a repetitive motor response pattern. There were two adaptation duration conditions: “adapt short” (200 ms) and “adapt long” (800 ms), each of which consisted of two blocks. Furthermore, there were two test color conditions in each block: “adapted color” (same as the adapting stimulus color) and “non-adapted color” (different from the adapting stimulus color). In total, there were four conditions. For each condition, participants completed 48 trials, including 40 trials with a test duration of 500 ms. Approximately, 10–12 min were required to complete each of the four blocks. After each block, there was a minimum 2-min break. All blocks and the trials in each block were completed in random order. Before the formal experiment, participants were required to complete several practice trials of the duration reproduction task.

### Results and Discussion

In Experiment 1, we focused only on the duration reproduced by participants when the duration of the test stimulus was 500 ms. To control for outliers, we applied a procedure similar to that used in previous studies (Li et al., 2017b). For each participant, all reproduction durations that were more than ±3 standard deviations from the participant’s mean reproduction duration for the corresponding condition (4.09% of all trials) were not included in further data analysis.

**Figure 2:**
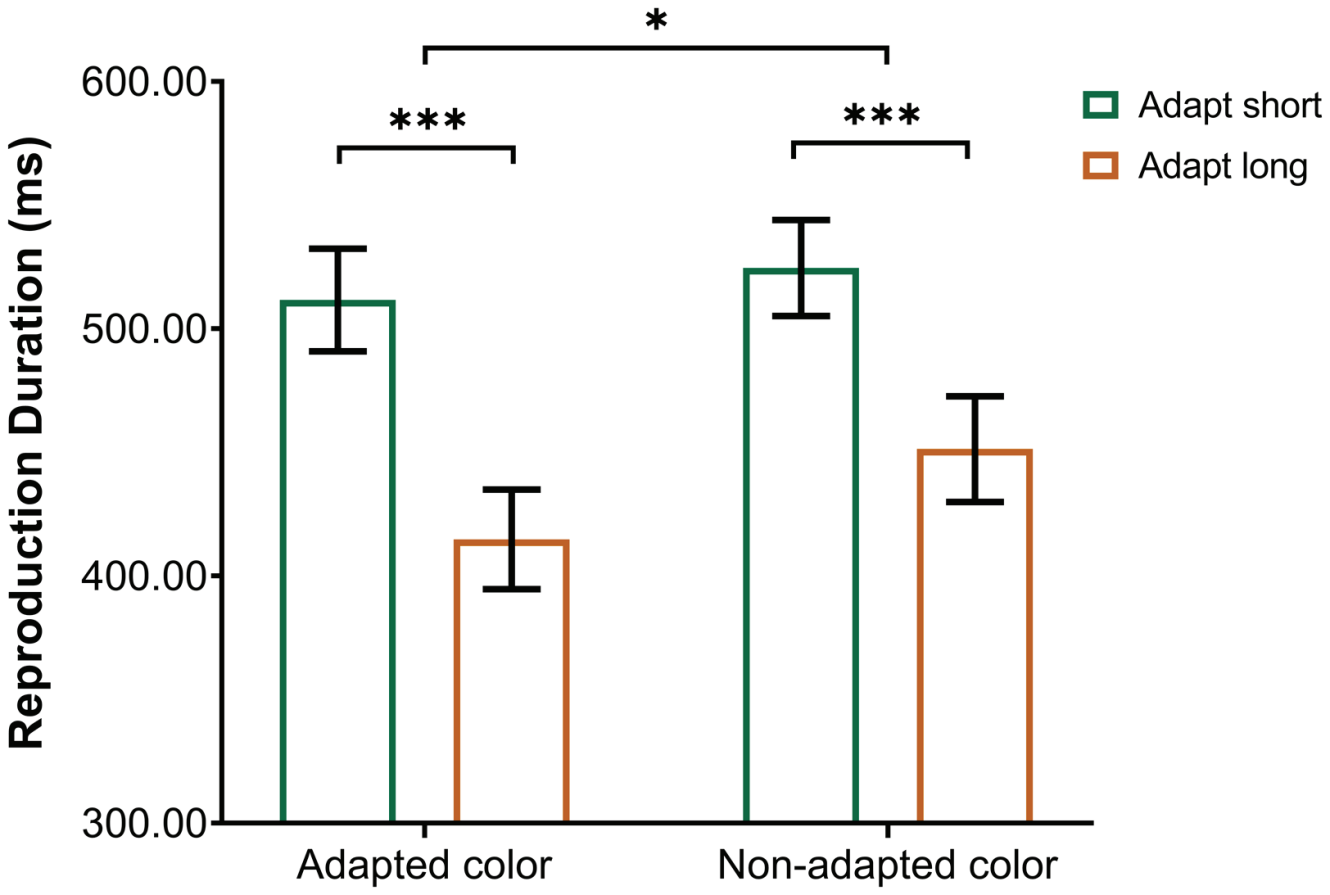
Interaction between the conditions of Experiment 1. Error bars represent standard errors. *p < 0.05, ***p < 0.001.

A repeated-measures ANOVA was performed on the remaining reproduction durations, with adaptation duration (adaptation short, adaptation long) and test color (adapted color and non-adapted color) as within-subject factors. The results revealed a significant main effect of adaptation duration [*F*(1,19) = 42.77, *p* < 0.001, *ηp*^2^ = 0.69] with longer duration reproduction following adaptation to a 200-ms stimulus compared to adaptation to an 800-ms stimulus. That is, adaptation to a shorter duration resulted in a longer perceived duration of the test stimulus compared to adaptation to a longer duration. In addition, we found a significant main effect of test color [*F*(1, 19) = 20.48, *p* < 0.001, *ηp*^2^ = 0.52], reflecting longer reproduced duration in the “non-adapted color” condition than in the “adapted color” condition. Importantly, there was a significant interaction between adaptation duration and test color [*F*(1,19) = 6.99, *p* < 0.05, *ηp*^2^ = 0.27]. Tests of simple effects showed that the reproduction duration of the “adapt short” condition (*M* = 511.62 ms, *SD* = 20.75) was significantly larger than that of the “adapt long” condition (*M* = 414.82 ms, *SD* = 20.12) in the “adapted color” condition (*p* < 0.001); the reproduction duration of the “adapt short” condition (*M* = 524.62 ms, *SD* = 19.45) was also significantly larger than that of the “adapt long” condition (*M* = 451.37 ms, *SD* = 21.36) in the “non-adapted color” condition (*p* < 0.001). To break down this interaction, contrasts were used to compare “adapted color” to “non-adapted color” and “adapt short” to “adapt long.” These contrasts revealed significant interactions when comparing “adapt short” to “adapt long” for “adapted color” compared to “non-adapted color” [*F*(1, 19) = 6.99, *p* < 0.05, *ηp*^2^ = 0.27]. As shown in the interaction diagram (Figure 2), these effects reflect that the reproduction duration of the “adapt short” condition was longer than that of the “adapt long” condition, and this difference was more pronounced for the “adapted color” than for the “non-adapted color” condition.

A measure similar to that used in previous studies was used to compare the aftereffect magnitude (Li et al., 2015a; Fulcher et al., 2016), which consisted of the arithmetic difference between the mean reproduction duration for the two adaptation conditions (duration aftereffect magnitude = mean reproduction duration of the “adapt short” condition—mean reproduction duration of the “adapt long” condition).

Specifically, the aftereffect magnitude in the adapted color or non-adapted color was the arithmetic difference between the mean reproduction duration of the white or red test stimulus in the “adapt short” and “adapt long” conditions.

**Figure 3:**
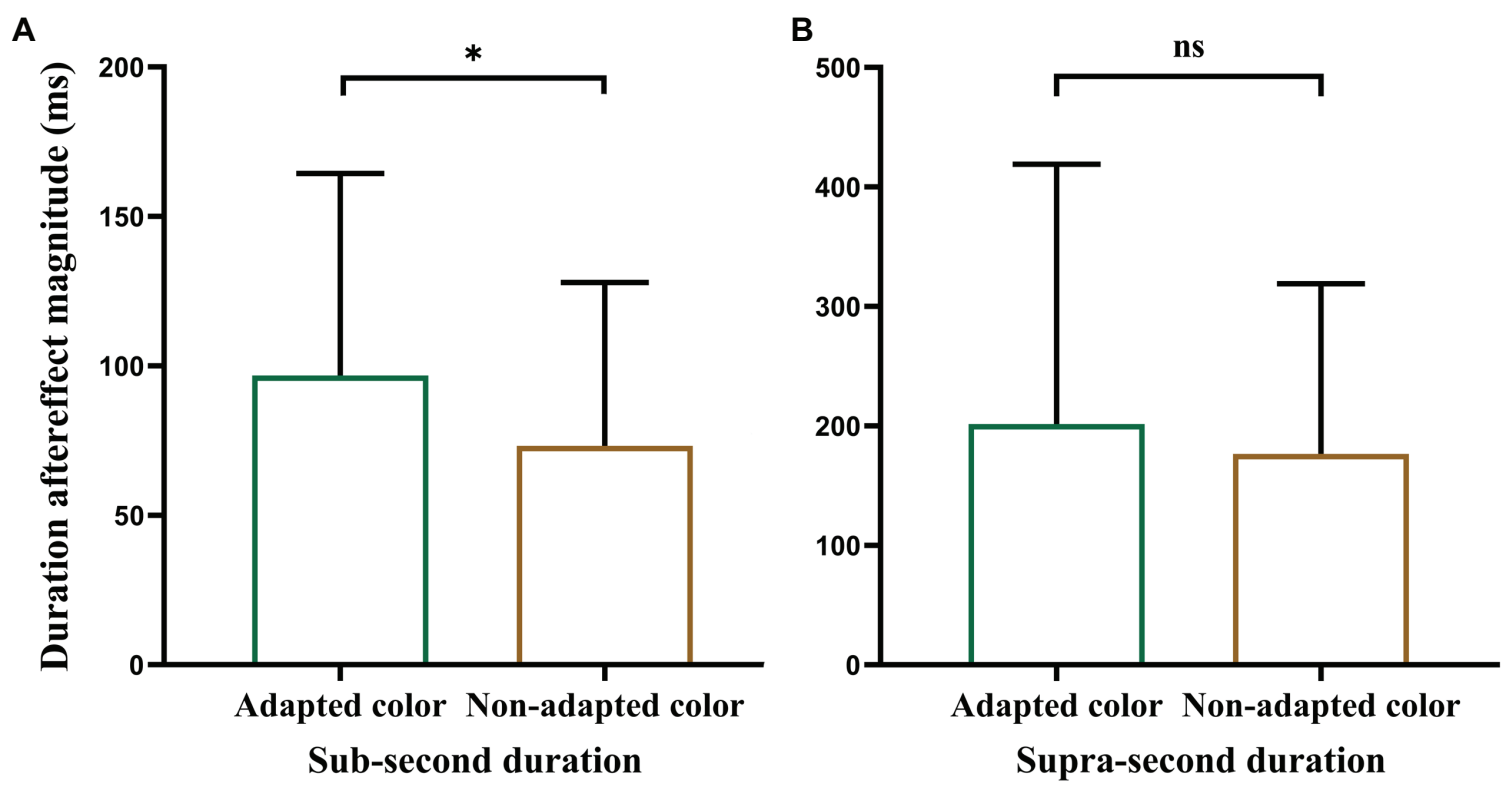
Plot of paired-samples *t*-test. **(A)** Refers to Experiment 1 and **(B)** to Experiment 2. Error bars represent standard errors. *p < 0.05.

Single-sample *t*-tests revealed that the aftereffect magnitudes were significantly larger than zero when the color of the test stimulus was the same as the adapted color (*M* = 96.80, *SD* = 67.57, *t* (19) = 6.41, *p* < 0.001) or non-adapted color (*M* = 73.26, *SD* = 54.68, *t* (19) = 5.99, *p* < 0.001). However, a paired-samples *t*-test (Figure 3A) revealed that the aftereffect magnitudes in the “adapted color” condition were significantly larger than those in the “non-adapted color” condition [*t* (19) = 2.64, *p* = 0.016]. These results suggest that, regardless of whether the color of the test stimulus was white or red, it produced a strong duration aftereffect in the sub-second range. However, the duration aftereffect in the sub-second range was only partly translation-invariant; that is, it partially transferred to the stimuli of different colors.

The results of Experiment 1 are consistent with those of previous studies, which found that duration adaptation occurs in the sub-second duration (Heron et al., 2012; Li et al., 2017a,b). The significant aftereffects for sub-second durations were observed not only when the color of the test stimulus was the same as that of the adaptation stimulus but also when it was different from the adaptation stimulus. Moreover, the results indicated that the duration aftereffect in the sub-second range could only partly transfer across different visual colors. In Experiment 2, we examined whether duration adaptation occurs in the supra-second duration and whether the aftereffect transfers across stimuli of different colors.

## Experiment 2

The aim of Experiment 2 was to investigate the color sensitivity of the duration aftereffect in the supra-second range.

### Materials and Methods

#### Participants

Experiment 2 included 21 participants (nine men, mean age: 21.10 ± 1.37 years) who were naïve to the experimental purpose and did not participate in Experiment 1. All participants were right-handed, had normal or corrected-to-normal vision, and provided written informed consent before the experiment.

#### Stimuli and Apparatus

The stimuli and apparatus used were the same as those in Experiment 1.

#### Procedure

The procedure was similar to that used in Experiment 1, except for the following changes. First, the durations of the adapting stimuli and test stimuli were greater than one second: the adapting durations were 1,500 ms (“adapt short” condition) and 4,000 ms (“adapt long” condition). However, the test stimulus durations varied randomly according to the following distribution: approximately 80% lasted 2,750 ms, approximately 10% lasted 2,000 ms, and the remaining 10% lasted 3,000 ms. Second, for each of the four conditions, participants completed 38 trials including 30 trials with a test duration of 2,750 ms. Approximately, 20–25 min were required to complete each of the four blocks. After each block, there was a minimum 2-min break. The participants completed the experiment over 2 days, with two blocks per day (one for 1,500 ms and the other for 4,000 ms). Both blocks and the trials in each block were completed in random order.

### Results and Discussion

In Experiment 2, we only focused on the duration reproduced by participants when the duration of the test stimulus was 2,750 ms. Using the same criteria as in Experiment 1, 3.85% of all trials were excluded from further analyses. Thereafter, using the same measure as in Experiment 1, the aftereffect magnitude of supra-second duration in each condition was calculated.

A 2 × 2 ANOVA for the reproduction durations of Experiment 2 revealed a statistically significant main effect of adaptation duration [*F*(1, 20) = 28.86, *p* < 0.001, *ηp*^2^ = 0.59], with longer duration reproduction in the “adapt short” condition (*M* = 2293.21 ms, *SD* = 72.62) than in the “adapt long” condition (*M* = 2103.99 ms, *SD* = 82.42). There was also a statistically significant main effect of test color [*F*(1, 20) = 4.67, *p* = 0.043, *ηp*^2^ = 0.19], with longer duration reproduction in the “non-adapted color” condition (*M* = 2222.31 ms, *SD* = 70.49) than in the “adapted color” condition (*M* = 2174.88 ms, *SD* = 81.97). However, the interaction was not significant [*F*(1, 20) = 0.42, *p* = 0.52, *ηp*^2^ = 0.02].

Single-sample *t*-tests revealed that aftereffect magnitudes were significantly larger than zero when the test stimulus color was the same as the adapted color (*M* = 201.66, *SD* = 217.37, *t* (20) = 4.25, *p* < 0.001) or non-adapted color (*M* = 176.77, *SD* = 142.32, *t* (20) = 5.69, *p* < 0.001). However, a paired-samples *t*-test (Figure 3B) revealed that there was no significant difference between the aftereffect magnitudes in the “adapted color” and “non-adapted color” conditions [*t* (20) = 0.65, *p* = 0.52]. These results suggest that, regardless of whether the color of the test stimulus was white or red, it produced a strong duration aftereffect in the supra-second range. Moreover, the duration aftereffect in the supra-second range was translation-invariant; that is, it could completely transfer to the stimuli of different colors.

In Experiment 2, consistent with previous studies (Shima et al., 2016; Li et al., 2017b), we found a significant aftereffect after adapting to supra-second durations, regardless of whether the color of the test stimulus was consistent with the adapted stimulus. Moreover, the results indicated that the duration aftereffect in the supra-second range could completely transfer across different visual colors. That is, the duration adaptation of supra-second duration showed color invariance.

## General Discussion

In the present study, we investigated the color sensitivity of the duration aftereffect in the sub-second or supra-second range. First, we provided further evidence that adaptation to stimuli of both sub-second and supra-second durations could induce duration aftereffects. Moreover, the duration aftereffect in the sub-second range could only partly transfer across different visual colors, whereas the supra-second duration aftereffect could completely transfer across different colors. That is, the color-sensitivity of the duration aftereffect in the sub-second duration was stronger than it was in the supra-second duration. The results of different color sensitivities of duration aftereffects in the sub-second and supra-second ranges suggest that the mechanisms underlying the adaptation aftereffects of the sub-second and supra-second ranges are distinct.

First, for the aftereffect produced by the sub-second duration, regardless of whether the color of the test stimulus was consistent with the adaptation stimulus, we observed a significant duration aftereffect. This is consistent with the results of most previous studies (Heron et al., 2013; Li et al., 2015a). Thereafter, for the aftereffect produced by the supra-second duration, we observed a significant duration aftereffect, regardless of the test stimuli’s colors. Although there are few studies of the duration aftereffect in the supra-second range, the results of previous studies concur that duration adaptation also occurs for supra-second durations (Shima et al., 2016; Li et al., 2017b).

Previous researches have studied the mechanisms of the duration aftereffect in the sub-second range. Heron et al. (2012) found that the sub-second duration aftereffect was sensory-specific, which indicated that the adaptation of the sub-second duration was occurred at a relatively early stage of visual and auditory sensory processing. Furthermore, Fulcher et al. (2016) found that the larger the adapting stimulus, the greater the spatial spread of the sub-second duration aftereffect, which indicated that the sub-second duration selective neurons pool spatial information across earlier stages of visual processing. Are the properties and mechanisms found in the studies of duration aftereffects in the sub-second range also applicable to aftereffects in the supra-second range? Our research answers this question by examining the color sensitivity of the duration aftereffect in the sub-second and supra-second ranges simultaneously. We found a difference between the color sensitivities of the duration aftereffects in the sub-second and supra-second ranges. Specifically, the duration aftereffect in the sub-second range could only partly transfer across different visual colors, whereas the duration aftereffect in the supra-second range could completely transfer across different visual colors.

When a stimulus is perceived, its color signals are transmitted along the ventral visual stream, from V1 to V2, V4, and ultimately to the inferior temporal cortex (Conway et al., 2010). Many neurons present at all levels of the visual cortex selectively respond to colors (Engel, 2005). Previous studies of color perception have shown that color-selective neurons tend to coincide with regions called blobs (Livingstone and Hubel, 1984); the color-responsive regions that are associated with blobs are joined by color “bridges” spanning adjacent blobs (Landisman and Ts’o, 2002). Therefore, a region of neurons responds when adapting to one color. The color-selective neurons that are consistent with adaptation have the strongest response, but other associated neurons may respond, albeit not as strongly. Moreover, neural recording studies have shown that the most color-selective neurons may relay color signals in V1 (Wachtler et al., 2003), and color adaptation research has found that later visual areas in the cortex may adapt more strongly than earlier visual areas (Engel, 2005). These studies of color selectivity support our results. For the sub-second duration, which involved greater processing in the early visual cortex, when individuals adapted to a white stimuli, the neurons selective for white may have exhibited the strongest response; neurons selective for red may have also responded, but their response was likely weaker. Therefore, we found that while both white and red stimuli had adaptive effects, the aftereffect for white stimuli was stronger than that for red stimuli. The results of the sub-second duration showed a partial transfer effect. For the supra-second duration that involved greater processing in the later visual cortex, the adaptation of color-selective neurons was stronger than that of the early visual cortex. Therefore, when individuals adapted to white stimuli, both white and red test stimuli had adaptive effects, and there was no difference in the aftereffects between the two. The supra-second duration results indicated a complete transfer effect. Our results show that mechanisms underlying the sub-second and supra-second duration aftereffects are different. The sub-second duration adaptation more involves the early visual cortex, while the supra-second duration adaptation more involves the later visual cortical regions. Meanwhile, the results also indicate that the duration aftereffects also exist for both low-level (early visual cortex) and high-level (later visual cortex) adaptations. Prior studies have confirmed this possibility; the coding of visual duration exists in multi-stages (Ivry and Schlerf, 2008; Merchant et al., 2013; Heron et al., 2019).

In the present study, we investigated the color sensitivity of the duration aftereffect in both the sub-second and supra-second ranges. Some important questions remain. Although the current study explores the mechanisms of the duration aftereffect of the sub-second and supra-second durations, the neural mechanisms underlying color perception are complex. The stimulus used in most previous studies of the duration aftereffect was a white Gaussian blob with a gray background. To facilitate comparison with previous results, we used white or red disks and a gray background. However, Mullen and Kingdom (2002) found that the two cone-opponent systems (red-green and blue-yellow systems) used by human color vision are functionally distinct. Recently, researchers found a surfeit of red and blue hue (end spectral of visible light) responses in V1 (Liu et al., 2020). Therefore, more differentiated colors should be considered in future research.

## Conclusion

The current study investigated the color sensitivity of the visual duration aftereffect in the sub-second and supra-second ranges. We found that the duration aftereffect in the sub-second range could only partly transfer across different visual colors, whereas the duration aftereffect in the supra-second range could completely transfer across different visual colors. The results show a difference between the color sensitivities of aftereffects produced by adapting to the sub-second and supra-second durations, which indicates that the mechanisms of duration aftereffect in sub-second and supra-second durations are different.

## Data Availability Statement

The raw data supporting the conclusions of this article will be made available by the authors, without undue reservation.

## Ethics Statement

The studies involving human participants were reviewed and approved by the Faculty of Psychology, Southwest University. The patients/participants provided their written informed consent to participate in this study.

## Author Contributions

BL: conceptualization, methodology, formal analysis, investigation, writing—original draft, writing—review and editing, and visualization. YC: conceptualization, methodology, formal analysis, writing—review and editing, and visualization. LP and GD: review and editing. XH: conceptualization, writing—review and editing, supervision, project administration, and funding acquisition. All authors contributed to the article and approved the submitted version.

## Conflict of Interest

The authors declare that the research was conducted in the absence of any commercial or financial relationships that could be construed as a potential conflict of interest.

## Publisher’s Note

All claims expressed in this article are solely those of the authors and do not necessarily represent those of their affiliated organizations, or those of the publisher, the editors and the reviewers. Any product that may be evaluated in this article, or claim that may be made by its manufacturer, is not guaranteed or endorsed by the publisher.

## References

[ref1] Battellil L. Walsh V. Pascual-Leone A. Cavanagh P. (2008). The ‘when’ parietal pathway explored by lesion studies. Curr. Opin. Neurobiol. 18, 120–126. doi: 10.1016/j.conb.2008.08.004, PMID: 18708141 PMC5076376

[ref2] Conway B. R. Chatterjee S. Field G. D. Horwitz G. D. Johnson E. N. Koida K.. (2010). Advances in color science: From retina to behavior. J. Neurosci. 30, 14955–14963. doi: 10.1523/jneurosci.4348-10.2010, PMID: 21068298 PMC3073527

[ref3] Engel S. A. (2005). Adaptation of oriented and unoriented color-selective neurons in human visual areas. Neuron 45, 613–623. doi: 10.1016/j.neuron.2005.01.014, PMID: 15721246

[ref4] Engel S. A. Furmanski C. S. (2001). Selective adaptation to color contrast in human primary visual cortex. J. Neurosci. 21, 3949–3954. doi: 10.1523/jneurosci.21-11-03949.2001, PMID: 11356883 PMC6762682

[ref5] Fulcher C. McGraw P. V. Roach N. W. Whitaker D. Heron J. (2016). Object size determines the spatial spread of visual time. Proc. R. Soc. B Biol. Sci. 283, 20161024. doi: 10.1098/rspb.2016.1024, PMID: 27466452 PMC4971211

[ref6] Hayashi M. J. Kantele M. Walsh V. Carlson S. Kanai R. (2014). Dissociable neuroanatomical correlates of subsecond and suprasecond time perception. J. Cogn. Neurosci. 26, 1685–1693. doi: 10.1162/jocn_a_00580, PMID: 24456398

[ref7] Heron J. Aaen-Stockdale C. Hotchkiss J. Roach N. W. McGraw P. V. Whitaker D. (2012). Duration channels mediate human time perception. Proc. R. Soc. B Biol. Sci. 279, 690–698. doi: 10.1098/rspb.2011.1131, PMID: 21831897 PMC3248727

[ref8] Heron J. Fulcher C. Collins H. Whitaker D. Roach N. W. (2019). Adaptation reveals multi-stage coding of visual duration. Sci. Rep. 9, 3016. doi: 10.1038/s41598-018-37614-3, PMID: 30816131 PMC6395619

[ref9] Heron J. Hotchkiss J. Aaen-Stockdale C. Roach N. W. Whitaker D. (2013). A neural hierarchy for illusions of time: duration adaptation precedes multisensory integration. J. Vis. 13, 1–12. doi: 10.1167/13.14.4, PMID: 24306853 PMC3852255

[ref10] Ivry R. B. Schlerf J. E. (2008). Dedicated and intrinsic models of time perception. Trends Cogn. Sci. 12, 273–280. doi: 10.1016/j.tics.2008.04.002, PMID: 18539519 PMC4335014

[ref11] Kastner S. Ungerleider L. G. (2000). Mechanisms of visual attention in the human cortex. Annu. Rev. Neurosci. 23, 315–341. doi: 10.1146/annurev.neuro.23.1.315 10845067

[ref12] Landisman C. E. Ts’o D. Y. (2002). Color processing in macaque striate cortex: relationships to ocular dominance, cytochrome oxidase, and orientation. J. Neurophysiol. 87, 3126–3137. doi: 10.1152/jn.2002.87.6.3126, PMID: 12037213

[ref13] Lewis P. A. Miall R. C. (2003a). Brain activation patterns during measurement of sub- and supra-second intervals. Neuropsychologia 41, 1583–1592. doi: 10.1016/S0028-3932(03)00118-0, PMID: 12887983

[ref14] Lewis P. A. Miall R. C. (2003b). Distinct systems for automatic and cognitively controlled time measurement: evidence from neuroimaging. Curr. Opin. Neurobiol. 13, 250–255. doi: 10.1016/s0959-4388(03)00036-9, PMID: 12744981

[ref15] Li B. Chen Y. Xiao L. Liu P. Huang X. (2017a). Duration adaptation modulates EEG correlates of subsequent temporal encoding. NeuroImage 147, 143–151. doi: 10.1016/j.neuroimage.2016.12.015, PMID: 27939922

[ref16] Li B. Xiao L. Yin H. Liu P. Huang X. (2017b). Duration aftereffect depends on the duration of adaptation. Front. Psychol. 8:491. doi: 10.3389/fpsyg.2017.00491, PMID: 28424646 PMC5380747

[ref17] Li B. Yuan X. Chen Y. Liu P. Huang X. (2015a). Visual duration aftereffect is position invariant. Front. Psychol. 6:1536. doi: 10.3389/fpsyg.2015.01536, PMID: 26500591 PMC4598571

[ref18] Li B. Yuan X. Huang X. (2015b). The aftereffect of perceived duration is contingent on auditory frequency but not visual orientation. Sci. Rep. 5:10124. doi: 10.1038/srep10124, PMID: 26054927 PMC4460570

[ref19] Liu Y. Li M. Zhang X. Lu Y. L. Gong H. L. Yin J. P.. (2020). Hierarchical representation for chromatic processing across macaque V1, V2, and V4. Neuron 108, 538.e5–550.e5. doi: 10.1016/j.neuron.2020.07.037, PMID: 32853551

[ref20] Livingstone M. S. Hubel D. H. (1984). Anatomy and physiology of a color system in the primate visual-cortex. J. Neurosci. 4, 309–356. doi: 10.1523/JNEUROSCI.04-01-00309.1984, PMID: 6198495 PMC6564760

[ref21] Maarseveen J. Hogendoorn H. Verstraten F. A. J. Paffen C. L. E. (2017). An investigation of the spatial selectivity of the duration after-effect. Vis. Res. 130, 67–75. doi: 10.1016/j.visres.2016.11.003, PMID: 27876514

[ref22] Meck W. H. (2005). Neuropsychology of timing and time perception. Brain Cogn. 58, 1–8. doi: 10.1016/j.bandc.2004.09.004, PMID: 15878722

[ref23] Merchant H. Perez O. Zarco W. Gamez J. (2013). Interval tuning in the primate medial premotor cortex as a general timing mechanism. J. Neurosci. 33, 9082–9096. doi: 10.1523/jneurosci.5513-12.2013, PMID: 23699519 PMC6705035

[ref24] Mullen K. T. Kingdom F. A. A. (2002). Differential distributions of red-green and blue-yellow cone opponency across the visual field. Vis. Neurosci. 19, 109–118. doi: 10.1017/s0952523802191103, PMID: 12180855

[ref25] Nieman D. R. Hayashi R. Andersen R. A. Shimojo S. (2005). Gaze direction modulates visual aftereffects in depth and color. Vis. Res. 45, 2885–2894. doi: 10.1016/j.visres.2005.06.029, PMID: 16095649

[ref26] Nishida S. Motoyoshi I. Andersen R. A. Shimojo S. (2003). Gaze modulation of visual aftereffects. Vis. Res. 43, 639–649. doi: 10.1016/s0042-6989(03)00007-5, PMID: 12604100

[ref27] Parkes L. M. Marsman J. B. C. Oxley D. C. Goulermas J. Y. Wuerger S. M. (2009). Multivoxel fMRI analysis of color tuning in human primary visual cortex. J. Vis. 9, 1.1–1.13. doi: 10.1167/9.1.1, PMID: 19271871

[ref28] Shima S. Murai Y. Hashimoto Y. Yotsumoto Y. (2016). Duration adaptation occurs across the sub- and supra-second systems. Front. Psychol. 7:114. doi: 10.3389/fpsyg.2016.00114, PMID: 26903920 PMC4746325

[ref29] Ungerleider L. G. (1995). Functional brain imaging studies of cortical mechanisms for memory. Science 270, 769–775. doi: 10.1126/science.270.5237.769 7481764

[ref30] Wachtler T. Sejnowski T. J. Albright T. D. (2003). Representation of color stimuli in awake macaque primary visual cortex. Neuron 37, 681–691. doi: 10.1016/s0896-6273(03)00035-7, PMID: 12597864 PMC2948212

